# Using Respondent Driven Sampling to Identify Malaria Risks and Occupational Networks among Migrant Workers in Ranong, Thailand

**DOI:** 10.1371/journal.pone.0168371

**Published:** 2016-12-29

**Authors:** Piyaporn Wangroongsarb, Jimee Hwang, Julie Thwing, Samart Karuchit, Suthon Kumpetch, Alison Rand, Chris Drakeley, John R. MacArthur, S. Patrick Kachur, Wichai Satimai, Sylvia Meek, David M. Sintasath

**Affiliations:** 1 Bureau of Vector Borne Diseases, Department of Disease Control, Ministry of Public Health, Nonathaburi, Thailand; 2 U.S. President’s Malaria Initiative, Malaria Branch, Division of Parasitic Diseases and Malaria, Centers for Disease Control and Prevention, Atlanta, Georgia, United States of America; 3 Global Health Group, University of California San Francisco, California, United States of America; 4 Malaria Branch, Division of Parasitic Diseases and Malaria, Centers for Disease Control and Prevention, Atlanta, Georgia, United States of America; 5 Thailand MOPH-US CDC Collaboration, Nonathaburi, Thailand; 6 Ranong Provincial Health Office, Ministry of Public Health, Ranong, Thailand; 7 Department of Infectious & Tropical Diseases, London School of Hygiene and Tropical Medicine, London, United Kingdom; 8 Malaria Consortium, London, United Kingdom; 9 U.S. President’s Malaria Initiative, USAID/RDMA, Bangkok, Thailand; Ehime Daigaku, JAPAN

## Abstract

**Background:**

Ranong Province in southern Thailand is one of the primary entry points for migrants entering Thailand from Myanmar, and borders Kawthaung Township in Myanmar where artemisinin resistance in malaria parasites has been detected. Areas of high population movement could increase the risk of spread of artemisinin resistance in this region and beyond.

**Methods:**

A respondent-driven sampling (RDS) methodology was used to compare migrant populations coming from Myanmar in urban (Site 1) vs. rural (Site 2) settings in Ranong, Thailand. The RDS methodology collected information on knowledge, attitudes, and practices for malaria, travel and occupational histories, as well as social network size and structure. Individuals enrolled were screened for malaria by microscopy, Real Time-PCR, and serology.

**Results:**

A total of 619 participants were recruited in Ranong City and 623 participants in Kraburi, a rural sub-district. By PCR, a total of 14 (1.1%) samples were positive (2 *P*. *falciparum* in Site 1; 10 *P*. *vivax*, 1 *Pf*, and 1 *P*. *malariae* in Site 2). PCR analysis demonstrated an overall weighted prevalence of 0.5% (95% CI, 0–1.3%) in the urban site and 1.0% (95% CI, 0.5–1.7%) in the rural site for all parasite species. PCR positivity did not correlate with serological positivity; however, as expected there was a strong association between antibody prevalence and both age and exposure. Access to long-lasting insecticidal treated nets remains low despite relatively high reported traditional net use among these populations.

**Conclusions:**

The low malaria prevalence, relatively smaller networks among migrants in rural settings, and limited frequency of travel to and from other areas of malaria transmission in Myanmar, suggest that the risk for the spread of artemisinin resistance from this area may be limited in these networks currently but may have implications for regional malaria elimination efforts.

## Introduction

Historically, resistance to anti-malarial drugs emerged first in the Greater Mekong Sub-region (GMS) to chloroquine, sulfadoxine-pyrimethamine (SP), and mefloquine, and population movements were partially responsible for the spread of the resistant parasites to other countries and regions [1,2]. Since the confirmation of artemisinin resistance along the Thailand-Cambodian border in 2009 [3,4], there has been concern about the risks of spread of artemisinin resistance to neighboring countries and increasing parasite clearance times to artemisinins have now been reported in Kawthaung, Myanmar [5], a township that shares an international border with Ranong, Thailand.

Ranong Province in southern Thailand is known to be one of the primary points of entry for migrants entering Thailand from Myanmar. Ranong historically has had both a high incidence of malaria and a high proportion of migrants (approximately 50% of residents are from Myanmar) compared to other Thai provinces. From the routine surveillance system, the annual parasite incidence (API) trend reported from Ranong Province was 12.0, 7.1, and 9.9 per 1,000 population in 2010, 2011, and 2012, respectively. By comparison, the national API was reported to be below 1 per 1,000 during this period.

Understanding the movement of migrant and mobile populations is essential to curb the potential spread of the resistant parasites, but the characteristics of this group make them inherently difficult to study. They are thought to be highly mobile, often hidden, and difficult to track with routine surveillance and to target with health interventions. Current standard cross-sectional household survey methods are inadequate to obtain representative information on this hidden, transient population due to the absence of an appropriate sampling frame. In an attempt to address some of these methodological issues, respondent-driven sampling (RDS) approach was adapted [6,7] as a potential tool to access these hard-to-reach populations.

Respondent-driven sampling is a modified chain-referral or snowball sampling technique used to approximate more precise estimates from hidden populations and has been used to study HIV risk groups [8] despite some methodological limitations [9]. Results from this quantitative survey, complemented with other qualitative information, should enable the Ministry of Public Health and its partners to understand better the behaviors and migration patterns of these populations, leading to enhanced surveillance and case management, and more effective targeting of malaria control interventions and health messages among migrant workers.

The aim of this study was to determine the migratory patterns, occupational risk, healthcare-seeking and malaria prevention behaviors, network associations, and parasite infection/exposure among mobile and migrant populations along the Thai-Myanmar border in an area with known artemisinin-resistant malaria parasites and to provide a reasonable sampling frame for estimates in these hard-to-reach populations. The need for such a survey was based on the underlying assumption that these mobile populations may represent a high-risk group and may contribute to the spread of the artemisinin resistant parasites, yet information on their malaria risk, migratory and network patterns have been limited.

In October 2009, an RDS study on migrant workers was carried out in Thailand along the Thai-Cambodia border and later a similar study was implemented in Cambodia to better understand internal migration patterns in two provinces on the border in the context of artemisinin resistance [10,11]. While there have been some studies along the northern stretch of the Thailand-Myanmar border [12–14], relatively little is known about migration along the southern portion of the border, which is believed to be a significant port of entry for many migrants from Myanmar. Furthermore, previous RDS studies in Thailand and Cambodia were only able to collect data on knowledge, attitude, and practices among migrant and mobile populations [11], and did not include collection of biological specimens. In this study, we screened all enrollees for malaria parasites and gathered information regarding their knowledge, attitudes, practices, and behaviors, as well as their network associations and propensity for travel.

## Methods

### Site selection

Two sites (urban and rural) were selected in Ranong Province, a southern province in Thailand that historically has had high malaria incidence and serves as an entry point for migrants from Myanmar. Initial recruits (seeds) were selected from migrant worker populations of Ranong City (urban) and Kraburi Sub-district (rural). Since there was not expected to be inter-mixing between the two study sites, sample size calculations were obtained for each site and sites were analyzed separately. With a migrant population proportion of 50%, design effect of 1.5, 95% confidence level, and a non-response rate of 10%, a total sample size of 600 participants was required for each study site.

Project staff recruited six seeds from each selected site for diversity in gender, age, and occupation. In total, 16 seeds were required (6 seeds for Site 1 and 10 seeds for Site 2) to reach the specified sample size. For Site 2 (rural), initial seeds were not able to recruit sufficient participants in a timely manner due to the remoteness of some locations and mobile teams were used to reach the desired sample size.

The inclusion criteria for the study included: 1) not being a Thai citizen; 2) coming to find work or economic advantage, or with someone who is; 3) age of at least 18 years; 4) no prior participation in this survey; and 5) provision of informed consent prior to enrollment. Pregnant women were not excluded as they were referred to receive nationally recommended treatment if found to be positive for malaria.

### Data collection and analysis

All enrolled participants were asked about their demographic background, migratory pattern, work history, health care seeking behavior including access and barriers to health messages, health status, and knowledge of malaria including its prevention. Data collection began on 1 May 2012 and ended on 15 July 2012 during the rainy season.

To minimize data collection errors, handheld personal digital assistants (HP iPAQ model HX 2007) with automatic data checks and skip patterns in the Myanmar language were used and the interviewers received training on the handling and use of these tools. Data were exported to Microsoft EXCEL. Following data cleaning and verification, analysis was performed using the Respondent-Driven Sampling Analysis Tool (RDSAT) Version 7.1.38 [15]. The data presented here are weighted based on reported individual network sizes using this tool.

### Biological screening

In order to assess current and previous malaria exposure, participants were asked to provide a blood sample for microscopy and filter paper collection to assess for current malaria infection using PCR and previous malaria exposure using serology. Reading of thick and thin blood smears was performed by local malaria staff and microscopy positive individuals were provided with antimalarial treatment according to the Thailand National Treatment Policy. All malaria positive slides and 10% of negatives were re-read at the National Reference Laboratory in Bangkok, Thailand. For molecular analyses, standard pooled, Real-Time PCR (RT-PCR) assays were used to differentiate plasmodia infections in dried blood spots [16]. Universal Safety Precautions were used in the collection, transport, storage, and analysis of biological specimens.

For serology, dried blood spot samples were eluted and assayed against antigens for both *P*. *falciparum* and *P*. *vivax* using methodologies previously described [17]. Briefly, antibody levels were determined by ELISA in Immulon4 96 well plates. Serum samples were added in duplicate at a concentration of 1/1000 for MSP-119 (*P*. *falciparum* and *P*. *vivax*), 1/1000 for MSP-2, 1/200 for CSP and 1/2000 for AMA (*P*. *falciparum* and *P*. *vivax*). Optical density was read at 492nm and antibodies reported as titers (as determined by standard on the plate).

### Ethical approval

Approval was obtained by the Ethics Committee for Research in Human Subjects of the Department of Disease Control, Ministry of Public Health, Thailand (FWA 00013622) and the US Centers for Disease Control and Prevention. Prior to enrollment in the study, participants provided written informed consent in Myanmar language. The inform consent form and procedures were also approved by the Ethics Committee.

## Results

### Demographics, occupational and travel history

A total of 619 participants were recruited in Ranong City (Site 1) and 623 participants in Kraburi sub-district (Site 2). Age distributions between the two sites were different, with proportionally younger participants in the rural site compared to the urban site (Table 1). The mean age of participants was 34.7 years (SD = 11.5) in the urban site (Site 1) compared to 30.8 years (SD = 11.0) in the rural site (Site 2). Gender distributions also differed significantly between the sites; more females were recruited in Site 1 (72%) than in Site 2 (42%). This could be due to the different types of work available in these two sites.

**Table 1:** RDS-weighted estimates of basic demographic characteristics of respondents by site.

		Site 1, Ranong City (N = 619)			Site 2, Kraburi (N = 623)		
		n	%	95% CI	n	%	95% CI
Sex	Male	177	28	23–33	361	58	54–64
	Female	429	72	67–77	246	42	36–46
Age group (years)	18–25	158	29	24–35	244	44	38–47
	26–35	199	32	27–37	196	30	26–35
	36–45	134	21	17–25	91	13	11–17
	46–70	115	18	14–22	76	13	11–17
Mean (SD) Age		34.7 (11.5)				30.8 (11.0)	
Ethnic group	Dawai	283	46	39–52	277	49	42–51
	Myanmar	251	43	38–51	238	40	38–46
	Mon	45	6	3–9	64	8	6–10
	Rhakine	4	1	0–1.4	15	2	1–4
	Karen	5	2	0–3	10	1	0–2
Able to speak	Myanmar	468	77	73–82	387	63	60–68
	Dawai	273	45	39–51	280	51	47–56
	Karen	3	1	0–1.3	10	1	0–2
	Mon	43	7	4–10	60	7	5–10
	Thai	247	33	28–37	-	-	-
Able to read	Myanmar	495	99	97–100	586	99	99–100
	Mon	11	-	-	45	5	4–7
	Karen	1	2	1–4	7	0.6	0–1
	Other	23	4	2–6	1	0	0–1
	Thai	32	7	4–11	-	-	-
Place of birth	Myanmar	591	98	96–99	606	99	99–100
	Thailand	11	2	1–4	1	0	0–0.5
Migrant status in Thailand	M1(≥6 months)	593	94	91–97	605	98	99–100
	M2(<6 months)	15	6	3–9	2	0.3	0–1

All participants were migrants from Myanmar. Migrants from other countries were not found in this study. Nearly all respondents in both sites were schooled in Myanmar (95%) and reported to be able to read Myanmar (>99%); although only 7% of migrants in Site 1 and none in Site 2 were able to read Thai. The most commonly used spoken languages among the respondents in both sites were Myanmar and Dawai. Nearly one-third of migrants in Site 1 were able to speak Thai. The majority of the respondents in both sites were long-term migrants classified as M1 (migrants living in Thailand for 6 months or more). Migrants in Site 1 reported having lived in Thailand on average 79.8 months compared to 61.6 months among migrants in Site 2. More than one-third of migrants in both sites had lived in Thailand for more than 5 years (Table 2).

**Table 2:** RDS-weighted estimates of travel and occupational history by site.

		Site 1, Ranong City (N = 619)			Site 2, Kraburi (N = 623)		
		n	%	95% CI	n	%	95% CI
Months in Thailand	1–5	15	6	3–9	2	0	0–1
	6–12	48	10	7–14	117	16	13–20
	13–60	260	48	43–53	292	50	46–55
	> 60	265	36	31–41	192	34	28–37
Plans to move to another place	Yes	34	5	3–7	100	20	15–23
Ever returned to Myanmar	Yes	229	33	28–38	146	22	19–29
	No	373	67	62–72	454	78	71–81
Frequency of return	>1x/mon	6	10	2–18	3	9	1–20
	>2x/yr	2	5	0–9	1	1	1–2
	1–2x/yr	13	15	5–27	34	44	32–62
	1x/2–3 yrs	35	33	22–52	5	41	27–55
	1x/5 yrs	21	24	15–41	1	4	1–6
	Never	10	13	2–22	-	-	-
Have work permit	Yes	342	91	87–95	401	68	65–75
	No	28	9	5–12	139	32	26–35
Type of permit	1 year	109	40	32–47	65	22	16–27
	Temp passport/visa	208	58	52–66	241	78	73–84
	Border pass	7	2	1–4	-	-	-
Why come to Thailand	No work in MYR	89	22	17–27	384	72	66–76
	Jobs irregular	79	22	18–29	236	39	35–45
	Get paid more	250	63	57–70	145	27	23–33
	Born here	4	1	0–2	6	2	1–3
	Persecution	1	1	0–1	1	0.4	0–0.6
Benefit from employer	Housing	35	10	6–13	542	99	99–100
	Water	4	1	0–2	462	87	82–91
	Land to farm	-	-	-	13	4	2–6
	Food	3	1	0–2	10	2	1–3
	Health insurance	227	58	50–63	3	1	0–2
Previous Industry	Agriculture	1	-	-	71	13	9–16
	Rubber plantation	4	1	0–3	436	79	75–83
	Domestic work	23	6	3–10	17	3	2–5
	Construction	63	15	11–20	20	5	3–8
	Factory	37	8	5–12	2	1	0–1.2
	Fishery	232	63	55–70	-	-	-
	Other	13	4	2–8	4	2	0–3
Duration	1–5 months	1	2	-	1	0	0–1
	6–12 months	26	19	-	71	20	17–27
	13–36 months	60	41	-	101	29	23–33
	>36 months	64	38	-	205	51	45–55
Industry currently working	Rubber plantation	-	-	-	441	77	72–82
	Palm plantation	-	-	-	67	14	9–16
	Domestic work	14	5	2–8	14	2	1–3
	Wood	47	10	7–15	25	5	3–9
	Fishery	218	61	54–69	-	-	-
	Factory	25	7	4–10	-	-	-
	Other	28	7	4–10	2	0	0–2

The primary reason for participants to come to Thailand was for work; more than half were assisted by relatives already living in Thailand. Very few migrants used middlemen to broker their trip to Thailand suggesting that most travel arrangements were done on an individual basis. One-fifth (20%) of those surveyed in Site 2 reported that they were planning to move to another location (mostly back to Myanmar) suggesting a more transient population in the rural site compared to the urban site (5%). Up to 33% and 22% of migrants from Site 1 and Site 2, respectively, had ever returned back to Myanmar. This may be due to the high cost of travel to return to Myanmar—the cost per trip reported by participants ranged from 2,000 to 300,000 Kyats (equivalent to $2.50 to $375 USD at the time of the study).

Differences were detected between Ranong city (Site 1) and Kraburi sub-district (Site 2) in terms of the occupational and residency profile of the migrants (Table 2). Migrants in the urban site were mostly associated with fisheries and those in the rural site predominantly worked on rubber plantations. Site 1 is more likely to have any type of work permit than Site 2, and more likely to have the more stable one-year permit (91%) compared to Site 2 (68%), where respondents were both less likely to have any kind of permit, and more likely to have only a temporary work visa. A majority of these migrants (72%) in Site 2 reported coming to work in Thailand because of the lack of work in Myanmar. Some migrants also cited other benefits received from their employers, including the provision of housing and health insurance.

### Knowledge, treatment-seeking, and health messages

Knowledge about malaria and how it is transmitted was quite high among the migrants in both sites (Table 3). While knowledge about the symptoms of malaria was generally acceptable with most respondents citing fever and chills to be associated with malaria, fevers as a sign of malaria were more often cited in urban Site 1 compared to rural Site 2. Consistent with malaria incidence data, very few respondents or family members had experienced a fever within the past 3 months in both sites.

**Table 3:** RDS-weighted estimates of malaria knowledge, exposure to health messages, and treatment-seeking behavior by site.

		Site 1, Ranong City (N = 619)			Site 2, Kraburi (N = 623)		
		n	%	95% CI	n	%	95% CI
Malaria transmission	Mosquito	368	74	69–79	407	94	91–96
	River water	85	16	13–20	126	35	31–42
	Rain	22	5	3–7	134	23	19–27
	Do not know	66	14	10–18	45	9	7–12
	Other insect	10	2	1–4	3	1	0–2
	Eating bananas	12	3	2–6	2	0	0–2
	Forest	86	16	12–20	-	-	-
Malaria symptoms	Chills	310	58	53–63	302	74	69–78
	Fever	371	75	71–80	153	33	28–38
	No appetite	18	4	2–7	11	24	19–28
	Sweat	26	5	3–7	42	8	6–11
	Cough	9	1	0–2	19	4	2–6
	Headache	134	27	23–34	97	24	19–29
	Do not know	87	18	14–22	-	-	-
Heard health message in last 3 months	Yes	210	33	29–38	102	19	16–23
Format of health message received	Radio	2	2	0–5	19	76	69–96
	Health education	76	66	46–73	17	71	52–86
	Interpersonal	9	7	2–15	7	23	6–42
	TV	13	18	10–36	2	10	0–61
	Brochure	64	68	60–81	2	11	0–16
	Billboards	40	41	27–51	1	3	0–11
Preferred format for health messages	Health worker	105	15	11–18	297	57	54–63
	Radio	12	1	0–2	320	49	45–54
	Interpersonal	14	3	1–5	181	35	32–41
	Migrant volunteers	422	64	59–68	53	7	5–10
	TV	77	16	12–20	20	4	3–6
	Billboard	152	26	21–30	1	3	2–4
	Brochures	284	47	42–53	2	1	0–2
Treatment seeking for last illness	Gov’t Hospital	323	50	44–55	548	87	-
	Private Hospital	98	16	12–20	-	-	-
	Malaria Post	-	-	-	1	0	0–1
	NGO	76	11	8–15	1	0	0–1
	Pharmacy	42	6	4–9	43	11	-
	Market/Shop	10	3	1–6	6	1	0–1.3
	CHW	1	0.2	0–1	4	0	0–1
	Self-treatment	8	2	0–3	1	0	0–1
	Nothing/nowhere	47	12	8–16	-	-	-
Country where treatment was sought	Thailand	486	99	97–100	536	89	86–92
	Myanmar	8	1	0–3	64	11	8–14
Why chose that location?	Closest	243	52	46–57	516	84	82–88
	Better quality	130	28	23–33	94	17	13–21
	Less expensive	49	12	8–16	9	3	0–5
	Treated better	23	5	2–7	99	13	11–17
	Health insurance	229	42	37–47	15	3	2–5
	Have translator	68	14	11–18	13	3	1–4

Only one third (33%) and 19% of migrants in Site 1 and Site 2, respectively, reported having heard a health message within past 3 months. Among those who received health messages in Site 1, the majority reported receiving those messages through health education by health workers (66%) and brochures (68%). In Site 2, the most common channels were through radio (76%) and health education by health workers (71%). Respondents in Site 1 preferred migrant volunteers (64%) and brochures (47%); whereas those in Site 2 preferred getting their information about health through health workers at facilities (57%) and the radio (49%). Malaria-related messages were more commonly heard in the rural Site 2 compared to the urban Site 1, where most health messages were about HIV/AIDS, STDs, and TB.

Treatment-seeking behaviors for fever among migrant populations were generally good. Half of the respondents in Site 1 and 87% in Site 2 reported having gone to a public government hospital for their last episode of fever (Table 3). In both sites, respondents did not report going to a malaria post for treatment of fever. A further 12% of respondents in Site 1 did not do anything for their fever; while 11% in Site 2 acknowledged purchasing drugs and treatments from local pharmacies. The main factors for choosing where to go for treatment of fever included proximity, better quality, and acceptance of health insurance.

### Malaria prevention

Ownership of at least one mosquito net was high in both sites (94% in Site 1 and 83% in Site 2) (Table 4). Among those who did not own a mosquito net, the most common reason cited was that they were not available or were too expensive.

**Table 4:** RDS-weighted estimates of malaria prevention by site.

		Site 1, Ranong City (N = 619)			Site 2, Kraburi (N = 623)		
		n	%	95% CI	n	%	95% CI
Number of mosquito nets owned	0	31	6	3–8	108	17	13–20
	1	247	39	35–44	460	78	75–82
	2	235	38	33–44	25	4	2–6
	3	81	15	11–18	8	1	0–2
	4	8	2	1–4	2	-	-
	5 or more	3	1	0–1	1	-	-
Owned at least one mosquito net	Yes	575	94	2–97	499	83	80–87
Type of net preferred	Conventional	288	59	54–65	76	20	16–25
	LLIN	237	41	35–46	333	82	76–86
	Hammock net	1	0	0–1	-	-	-
Usual working time	Day	444	85	80–88	78	20	16–25
	Night	5	1	0–2	327	79	74–83
Slept under a net last night (among net owners)	Yes	476	90	87–93	404	99	97–100
Slept under an LLIN	Yes	3	1	0–1	8	2	1–5
Net obtained from?	**Free**						
	Employer	4	1	0–2	16	4	2–5
	Health facility	-	-	-	8	3	1–5
	CHW	-	-	-	6	1	0–2
	Family/friends	42	11	7–15	-	-	-
	**Bought**						
	Employer	4	2	1–5	2	1	0–1
	Health facility	1	0	0–1	6	1	0–3
	CHW	1	1	0–1	2	1	0–1
	Family/friends	8	2	1–4	-	-	-
	Market/Shop	374	84	79–88	367	93	90–95

Two-thirds of respondents in Site 1 preferred to use conventional untreated nets; whereas an overwhelming majority (82%) of respondents in Site 2 preferred to use long lasting insecticidal treated nets (LLINs). Although not specifically probed, this difference could be due to the repellency effect of the LLIN.

Migrant workers in the urban site generally were employed during the day, while those in the rural site were generally working at night, mostly in the rubber plantations. Nearly all migrant workers in Site 2 reported having slept the previous night in housing structures or sleeping areas provided by their employers and having slept under a mosquito net. However, most of these mosquito nets were not LLINs even though there was a strong preference for LLINs and most mosquito nets had been purchased from the shops or market in both sites.

### Malaria prevalence

Although the prevalence of malaria detected during the period of the survey was found to be very low in both sites, there was marginal statistical association (p = 0.054) between having traveled back to Myanmar in the past 12 months and serological positivity among respondents in Site 1, but not statistically different among respondents in Site 2 (p = 0.216) using multi-variate logistic regression analysis. By double-read microscopy, only one (1) *P*. *vivax* positive case was detected in an individual from Site 2. This individual was a 48-year old female from the Dawai ethnic group, who had lived in Thailand for 3 years, and had traveled back to Myanmar once or twice per year. However, this individual was found to be both PCR and serologically confirmed negative, which might suggest to the possibility of a false positive slide reading despite double cross-checking.

By PCR, 14 samples were found to be positive (2 individuals in Site 1 and 12 individuals in Site 2) (Table 5). The majority of cases were *P*. *vivax* (10/14) with two *P*. *falciparum*, one mixed *P*. *falciparum* and *P*. *vivax*, and one *P*. *malariae* infections. PCR analysis demonstrated an overall weighted prevalence of 0.5% (95% CI, 0–1.3%) in the urban site (Ranong City) and 1.0% (95% CI, 0.5–1.7%) in the rural site (Kraburi) for both parasite species—although the predominant species was *P*. *vivax*, which presents a limited risk for spread of artemisinin resistance and to date has only been identified in *P*. *falciparum*.

**Table 5:** Summary of individuals with positive blood film, PCR, and/or serology.

Individual	Site	Age	Sex	Ethnicity	Travel to Myanmar	Blood Slide	PCR	Serology
1	1	28	F	Myanmar	Yes	Negative	*P*. *falciparum*, *P*. *vivax*	Negative
2	1	25	F	Dawai	No	Negative	*P*. *falciparum*	Negative
3	2	19	F	Dawai	No	Negative	*P*. *vivax*	Negative
4	2	36	F	Dawai	No	Negative	*P*. *vivax*	Negative
5	2	38	F	Mon	No	Negative	*P*. *vivax*	*P*. *falciparum*, *P*. *vivax*
6	2	30	M	Myanmar	No	Negative	*P*. *vivax*	*P*. *falciparum*
7	2	30	M	Myanmar	No	Negative	*P*. *vivax*	Negative
8	2	27	F	Myanmar	Yes	Negative	*P*. *vivax*	*P*. *falciparum*, *P*. *vivax*
9	2	40	M	Dawai	No	Negative	*P*. *vivax*	*P*. *vivax*
10	2	30	F	Dawai	No	Negative	*P*. *malariae*	*P*. *falciparum*, *P*. *vivax*
11	2	48	F	Dawai	Yes	*P*. *vivax*	Negative	Negative
12	2	18	F	Myanmar	No	Negative	*P*. *vivax*	*P*. *vivax*
13	2	41	M	Myanmar	No	Negative	*P*. *falciparum*	*P*. *falciparum*, *P*. *vivax*
14	2	21	M	Myanmar	No	Negative	*P*. *vivax*	*P*. *vivax*
15	2	27	F	Dawai	No	Negative	*P*. *vivax*	Negative

**Fig 1:**
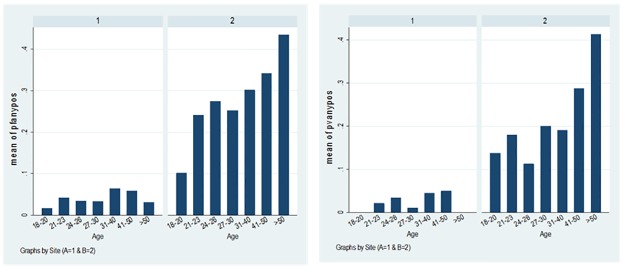
Mean of seropositivity to any tested antigens for *P*. *falciparum* (left) and *P*. *vivax* (right) by age groups.

**Fig 2:**
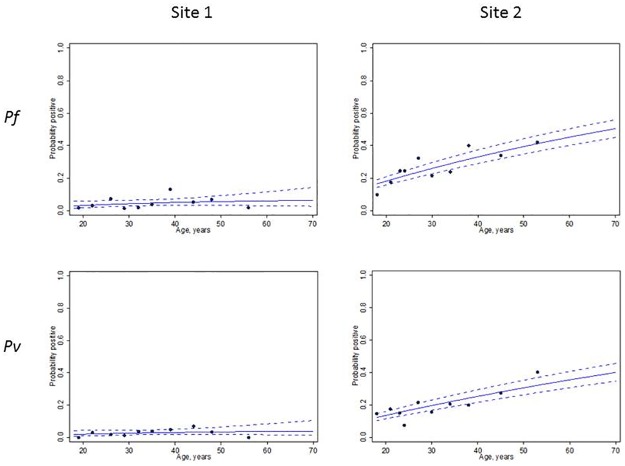
*P*. *falciparum* (upper boxes) and *P*. *vivax* (lower boxes) seroprevalence curves for each site. Circles represent actual data points (placed at percentiles, solid blue line is the maximum likelihood fitted model and dotted lines represent 95% CI for the model).

There were significant differences in serological profiles between the two sites (Table 6). The population in Site 2, a rural site with proximity to the forest fringes, had higher serological reactivity to *P*. *falciparum* (24%) and *P*. *vivax* (15%) antigens, compared to the urban site (4% *P*. *falciparum* and 3% *P*. *vivax*). Serological reactivity to either *P*. *falciparum* or *P*. *vivax* antigens among the population in Site 2 was 31% compared to 7% in Site 1. Particularly in Site 2, there was a clear association between increasing seroprevalence and age strongly suggesting that seroprevalence may be cumulatively representing current and past exposure (Fig 1). Furthermore, serological response appeared higher to each combined antigen implying differential responsiveness and supporting the use of multiple antigens for serological screening in low transmission settings. Seroconversion rates represent the rate at which the population becomes antibody positive to specific malarial antigens. Assuming a constant rate of reversion, measurable differences in exposure to both *P*. *falciparum* and *P*. *vivax* between the two study populations can be observed (Fig 2).

**Table 6:** RDS-weighted estimates comparing biological results by site.

		Site 1, Ranong City (N = 619)			Site 2, Kraburi (N = 623)		
		n	%	95% CI	n	%	95% CI
PCR (*P*. *falciparum* or *P*. *vivax*)	Positive	2	0.5	0–1.3	12	1	0.5–1.7
	Negative	606	99.5	98.7–100	595	99	98–100
Serological profile	*P*. *falciparum*	26	4	2–6	157	24	21–29
	*P*. *vivax*	16	3	1–5	118	15	13–18
	Either *P*. *falciparum* or *P*. *vivax*	37	7	4–9	201	31	28–37

### Network associations

One of the key aspects of the RDS methodology was the determination of network patterns among the respondents. In Site 1, most of the recruiters were either friends (47%) or neighbors (46%) of the respondent; whereas, in Site 2, a majority of the recruiters were identified as employers (Table 7). The majority of respondents in both sites had relatively modest network sizes. That is, each respondent in Site 1, on average, reported having seen or interacted with 17 other migrants during the past week; whereas, the average was 20 migrants per week for Site 2. Personal interest in this research was cited as the most common reason for joining the study in both sites. Analysis of migrants in Site 1 who have been in Thailand for 6 months or more (M1) compared to those who have been in Thailand for less than 6 months (M2) shows that M1 migrants with an average network size of 10.4 tended to be more homophilous (Hx = 0.552)–that is, they preferred to associate more within their own established networks. On the other hand, M2 migrants with a smaller average network size of 4.4, exhibited strong heterophily (Hx = -1.0) (or tendency to not associate within their own networks).

**Table 7:** RDS-weighted estimates of network characteristics by site.

		Site 1, Ranong City (N = 619)			Site 2, Kraburi (N = 623)		
		n	%	95% CI	n	%	95% CI
Relationship with recruiter	Employer	1	0	0–1	478	76	68–84
	Friend	294	47	42–52	118	22	16–30
	Coworker	66	10	7–13	52	7	5–10
	Husband/wife	-	-	-	6	1	0–2
	Stranger	8	2	1–4	1	0	0–0.4
	Other relative	3	1	0–1	-	-	-
	Neighbor	281	46	41–51	-	-	-
Age of recruiter	18–25	116	20	16–24	69	16	10–21
	26–35	286	44	39–49	91	15	12–19
	36–45	149	25	21–30	106	15	11–18
	46–70	56	10	7–14	339	54	48–63
Mean (SD) Age		33.8 (8.5)			45.7 (13.0)		
Know recruiter (months)	1–5 months	51	13	9–18	22	4	2–6
	6–12 months	152	29	24–34	151	25	20–28
	13–60 months	287	44	38–49	286	43	38–48
	> 60 months	117	14	11–17	146	29	24–34
Frequency of meeting recruiter	Daily	426	69	65–74	313	52	-
	Several times/week	137	22	17–26	203	32	-
	Once per week	33	7	5–10	61	11	8–17
	Once per month	7	1	0–2	15	2	1–5
	< 1 per month	4	1	0–3	13	3	1–5
Number of other migrants seen last week	0	1	0	0–1	1	1	0–4
	1–10	293	75	71–78	35	55	43–63
	11–20	222	21	18–25	37	28	21–40
	21–50	82	4	3–5	33	16	12–21
	>50	6	0	0–1	-	-	-

## Discussion

Respondent-driven sampling has been used for nearly 20 years for the sampling of hard-to-reach populations such as intravenous drug users and commercial sex workers [18,19]. This methodology has matured to serve as the basis for the surveillance of HIV/AIDS and other biological markers [20]. More recently, RDS was adaptively used along the Thailand-Cambodia border to study migrant populations [10,11] that could contribute to spreading artemisinin resistant malaria parasites throughout the GMS. Despite the advantages of being able to sample from such hidden, hard-to-reach populations, there are challenges with sampling errors, statistical inferences, and wide confidence intervals that should be kept in mind when interpreting data from RDS [21]. This innovative sampling technique aims to provide stable estimates for populations lacking adequate sampling frames, but is not without its drawbacks. Biases can be introduced from the high variance of estimates and fairly narrow confidence intervals obtained from the RDS methodology. Discussed in detail elsewhere [9], these issues should be considered in the interpretation of results. Despite these statistical limitations, however, the operational application of RDS as a means for data collection and biological screening of hard-to-reach populations could make this an attractive tool for national programs seeking representative evaluation data.

Accessing migrant populations is intrinsically difficult for public health programs, particularly those in the GMS, where borders are porous and often where illicit forest-related activities occur. Furthermore, migrant populations who are unregistered and without work permits or have entered the country illegally often will avoid government facilities for fear of being caught, which makes accessing these populations that much more difficult. The majority of migrant workers in both study sites have work permits and were likely more amendable to enrolling in this study. Developing innovative and effective methods of reaching migrant populations was identified as a priority for responding to artemisinin resistance [22], and ultimately for the elimination of malaria [23].

To our knowledge, this is the first use of RDS to collect biological specimens for malaria from cross-border migrant populations. More than 1,200 migrants from Myanmar who are living and working in urban and rural sites in Thailand were screened using standard microscopy, q-PCR, and serological assays for the detection of malaria infection, previous exposure, and assessed for their access to malaria prevention and treatment services. Malaria prevalence detected through microscopy yielded only one *P*. *vivax* malaria case, raising the question whether microscopy alone is sufficient in such low transmission settings.

To ascertain previous malaria infection, serology has been proposed as a useful way for estimating exposure to malaria parasites in low transmission settings [24–26]. Serological analyses using antigen-specific assays for both *P*. *falciparum* and *P*. *vivax* yielded an interesting distribution of seropositivity when comparing between the urban and rural sites. Sero-reactivity to *P*. *falciparum* antigens ranged from 4% (2%-6%) in Site 1 compared to 24% (21%-29%) in Site 2, suggesting not surprisingly, significantly higher risks of *P*. *falciparum* infection in those residing in the rural site compared to the urban site. Similar patterns of sero-reactivity to *P*. *vivax* antigens were also observed between the two sites. These results suggest that the rural site, although having only one parasitemic individual identified through gold-standard microscopy at the time of this cross sectional survey, may still experience substantial seasonal malaria transmission as evidenced by the prevalence of antibodies in these relatively stationary migrant populations. Additional investigations may be needed to determine whether these individuals were exposed and infected in Myanmar or in Thailand, though the data suggest that cross border movement occurs less frequently in this population than previously expected. Furthermore, in Site 1, the two malaria cases detectable by PCR were *P*. *falciparum* and serologically negative; whereas in Site 2, most of the PCR positive cases were *P*. *vivax* with a much more diverse serological profile, suggesting greater exposure to malaria antigens over time.

Most migrants in Ranong Province are long-term residents and have not traveled frequently back to Myanmar. According to the definition used in Thailand, these migrants would be considered M1 migrants (those who have lived in Thailand for 6 months or more). Migrants in both sites had access to and reported use of mosquito nets; however, the vast majority of these were conventional, untreated mosquito nets that were purchased in the markets. The current net culture that exists among these migrant populations is encouraging, and strategies (e.g., employer-based distribution) should be considered to replace and/or convert these conventional nets into more effective LLINs that could have greater impact on the reduction of malaria transmission. Furthermore, three-quarters of those in Site 2 were rubber tappers and alternative outdoor personal protection methods (e.g., insecticide-treated clothing) should also need to be considered.

Examination of a group’s tendency to associate within or outside one’s own networks, average network sizes, and social network analyses offer insights into the potential use of influential individuals to promote health and treatment-seeking behaviors. This study highlights that the network sizes of migrants in urban Ranong are larger and more homophilous (i.e., tendency to associate within their own established networks) compared to migrants in the rural setting, who tend to have smaller network sizes and stronger affiliations with their employers. Using RDS to better understand the social and occupational networks of migrants in different settings could help malaria programs better target delivery of malaria services such as distribution of LLINs, health education and promotion, or even routine screening and treatment of migrant populations. The benefits of identifying migrants who otherwise would not have been reached through routine public services, and providing health care services for these individuals are worth considering. Furthermore, analysis of networks and mobility patterns through the use of RDS can contribute valuable epidemiological and service utilization data from these hard-to-reach populations which is increasingly critical for malaria surveillance in the context of elimination.

There were challenges and limitations that may have affected the outcome of this study. Firstly, relatively high transportation costs and long travel distances to the enrollment site may have affected recruitment of participants in the more rural site. Measures were put in place to mitigate these barriers, including moving the recruitment sites closer to the migrants wherever possible. Secondly, there may be a risk of convenience sampling occurring if coupons were not distributed through networks. Therefore, the selection of seeds is critical [27] and should be reflective of the target population profile. It should be noted that the migrant workers in this study were relatively accessible and may not be representative of the migrant population in Ranong. Thirdly, as with cross-sectional surveys, measuring parasitemia through the use of RDS only provides a snap-shot of the prevalence and longitudinal monitoring may be needed to better understand malaria transmission trends. Lastly, formative research must inform the appropriateness of the use of RDS or snowball sampling to where social networks exist within the population of interest.

The low prevalence of malaria parasitemia, relatively homogenous networks among these migrants, and limited frequency of travel to and from other areas of malaria transmission, specifically Myanmar, all suggest limited potential for spread of artemisinin resistance along the Kawthaung-Ranong corridor through these populations but the frequency and extent of population mobility in this region can be variable. Described here, RDS can be a potential programmatic tool to obtain reasonably representative estimates among migrant populations, to better understand different migrant networks and behaviors, and ultimately to improve access to and delivery of malaria services to these hard-to-reach populations and their associated networks.
